# Prevalence and predictors of infant and young child feeding practices in sub-Saharan Africa

**DOI:** 10.1093/inthealth/ihad022

**Published:** 2023-04-12

**Authors:** Richard Gyan Aboagye, Abdul-Aziz Seidu, Bright Opoku Ahinkorah, Abdul Cadri, James Boadu Frimpong, Louis Kobina Dadzie, Eugene Budu, Oghenowede Eyawo, Sanni Yaya

**Affiliations:** Department of Family and Community Health, Fred N. Binka School of Public Health, University of Health and Allied Sciences, Hohoe, Ghana; Department of Population and Health, University of Cape Coast, Cape Coast, Ghana; College of Public Health, Medical and Veterinary Sciences, James Cook University, Australia; Centre for Gender and Advocacy, Takoradi Technical University, Takoradi, Ghana; School of Public Health, Faculty of Health, University of Technology Sydney, Sydney, NSW, Australia; Department of Social and Behavioural Science, School of Public Health, University of Ghana, Legon, Ghana; Department of Family Medicine, Faculty of Medicine, McGill University, Montreal, QC, Canada; Department of Health, Physical Education, and Recreation, University of Cape Coast, Cape Coast, Ghana; Department of Kinesiology, New Mexico State University, Las Cruces, NM, USA; Department of Population and Health, University of Cape Coast, Cape Coast, Ghana; Department of Population and Health, University of Cape Coast, Cape Coast, Ghana; School of Global Health, Faculty of Health, York University, Toronto, ON, Canada; School of International Development and Global Studies, University of Ottawa, Ottawa, ON, Canada; George Institute for Global Health, Imperial College London, London, UK

**Keywords:** children, complementary feeding, global health, infant feeding, sub-Saharan Africa, women

## Abstract

**Background:**

This study assessed the prevalence and predictors of minimum dietary diversity (MDD), minimum meal frequency (MMF), and minimum acceptable diet (MAD) in sub-Saharan Africa (SSA).

**Methods:**

A sample of 87 672 mother–child pairs from the 2010–2020 Demographic and Health Surveys of 32 countries in SSA was used. Multilevel binary logistic regression analysis was carried out to examine the predictors of MDD, MMF, and MAD. Percentages and adjusted odds ratios (aORs) with a 95% confidence interval (CI) were used to present the findings.

**Results:**

The prevalence of MDD, MMF, and MAD in SSA were 25.3% (95% CI 21.7 to 28.9), 41.2% (95% CI 38.8 to 43.6), and 13.3% (95% CI 11.6 to 15.0), respectively. Children aged 18–23 months were more likely to have MDD and MAD but less likely to have MMF. Children of mothers with higher education levels were more likely to have MDD, MMF, and MAD. Children who were delivered in a health facility were more likely to have MDD and MAD but less likely to have MMF.

**Conclusions:**

Following the poor state of complementary feeding practices for infants and young children, the study recommends that regional and national policies on food and nutrition security and maternal and child nutrition and health should follow the internationally recommended guidelines in promoting, protecting, and supporting age-appropriate complementary foods and feeding practices for infants and young children.

## Introduction

The United Nations Convention on the Rights of the Child stated that every child has the right to good nutrition.^1^ This implies that good nutrition should not be a privilege, but is a necessity for every child. However, this right is yet to be met for many children, as the World Health Organization (WHO) reported that in 2020, about 149 million children <5 y of age were estimated to be stunted (too short for their age), 45 million were wasted (too thin for their height), and 38.9 million were overweight or obese.^2^ It has been reported that few children receive nutritionally adequate and safe complementary foods, emphasizing that in most countries, less than a quarter of infants aged 6–23 months met the criteria for dietary diversity and feeding frequency that is appropriate for their age.^3^ Although undernutrition is associated with 45% of child mortality globally, the lives of >820 000 children <5 y of age could be saved yearly if they are fed appropriately.^2,3^

The infant and child mortality rate remains high in low-and middle-income countries (LMICs), especially in sub-Saharan Africa (SSA), and nutrition-related causes account for a substantial proportion of these mortalities.^4–6^ It is worth noting that a key determinant of the nutritional status of young children or infants in SSA is their feeding practices.^7,8^ WHO has several indicators for measuring infant and young child feeding practices. These include minimum dietary diversity (MDD), minimum meal frequency (MMF), and minimum acceptable diet (MAD).^9^

MDD is the proportion of children 6–23 months of age who receive foods from a minimum of five of the eight groups of foods, namely breast milk, grains, roots and tubers; legumes and nuts; dairy products; flesh foods (meat, fish, poultry and organ meats); eggs; vitamin A–rich fruits and vegetables; and other fruits and vegetables.^10,11^ MMF is the proportion of children 6–23 months of age who receive solid, semisolid or soft foods (but also including milk feeds for non-breastfed children) the minimum number of times or more (two feedings of solid, semisolid or soft foods for breastfed infants ages 6–8 months; three feedings of solid, semisolid or soft foods for breastfed children ages 9–23 months; and four feedings of solid, semi-solid or soft foods or milk feeds for non-breastfed children ages 6–23 months, whereby at least one of the four feeds must be a solid, semisolid or soft food).^10,12^ Lastly, MAD refers to receiving at least the MDD and MMF for their age during the previous day for breastfed children and for non-breastfed children receiving at least the MDD and MMF for their age during the previous day as well as at least two milk feeds.^10,13^

The feeding practices of infants and young children in SSA are very important because inappropriate feeding practices have been reported to increase the risk of undernutrition, micronutrient deficiencies, morbidities, mortalities, and nutrition-related non-communicable diseases.^14–16^ These inappropriate feeding practices account for more than two-thirds of child and infant mortality in SSA.^17^ Children who do not receive sufficient dietary diversity, meal frequency or acceptable diet after 6 months of age are at a higher risk of becoming stunted, despite having been optimally breastfed.^11^ Moreover, the findings of Masuke et al.^17^ indicated that inappropriate feeding practices are associated with a higher risk of stunting, wasting, and underweight among young children. Also, Yarnoff et al.^18^ reported that inappropriate feeding practices are associated with diarrhoea, fever and cough among infants and young children in SSA. The quality of feeding practices in infants and young children is dependent on the frequency of the meal, the food groups contained in the diet and the acceptability of the diet. However, most infants and young children are introduced to normal household foods, which are predominantly cereals and starchy foods, and are poor in quality.^19,20^

The 2014 report of the International Food Policy and Research Institute on good global nutrition reported that to achieve nutritional requirements and prevent deficiencies, child feeding practices in the first 2 y of age need special attention, especially in SSA.^21^ There have been some studies conducted on infant and young child feeding practices in SSA,^22,23^ but these studies have limited generalizability due to the small sample sizes and different methods of assessment. The present study sought to fill the gaps in the literature accordingly. This study aimed at assessing the prevalence of MDD, MMF, and MAD in SSA at the subregional level, as well their predictors. The findings of this study will provide evidence to improve the dietary practices of infants and young children in SSA in line with the Sustainable Development Goal 3.2.^24^

## Methods

### Study design and data description

Our study was multicountry and involved a cross-sectional data analysis of the Demographic and Health Surveys (DHS) conducted from 2010 to 2020 in 32 sub-Saharan African countries. The data were extracted from the children's file (KR) of the 32 countries. The DHS is a nationally representative and comparative survey conducted every 5 y in >90 low- and middle-income countries (LMICs) globally.^25^ Respondents for the survey were selected using a two-stage cluster sampling technique. The detailed sampling procedure has been published elsewhere.^26^ The DHS employed a standardized, structured questionnaire to collect data from respondents on health indicators such as child nutrition and feeding practices.^25^ In the present study, we included 87 672 mother–child pairs in the final analysis (see Table 1). The dataset for the 32 countries used is freely available from https://dhsprogram.com/data/available-datasets.cfm.

**Table 1.** Description of the study

Countries	Year of survey	Weighted N	Weighted %
1. Angola	2015–16	3511	4.0
2. Burkina Faso	2010	4034	4.6
3. Benin	2017–18	3773	4.3
4. Burundi	2016–17	3975	4.5
5. Congo DR	2013–14	4746	5.4
6. Congo	2011–12	2402	2.7
7. Cote d'Ivoire	2011–12	2045	2.3
8. Cameroon	2018	2616	3.0
9. Ethiopia	2016	2942	3.4
10. Gabon	2012	1349	1.5
11. Ghana	2014	1646	1.9
12. Gambia	2019–20	2121	2.4
13. Guinea	2018	1786	2.0
14. Kenya	2014	2584	2.9
15. Comoros	2012	712	0.8
16. Liberia	2019–20	1335	1.5
17. Lesotho	2014	920	1.0
18. Mali	2018	2694	3.1
19. Malawi	2015–16	4720	5.4
20. Nigeria	2018	8722	9.9
21. Niger	2012	3363	3.8
22. Namibia	2013	966	1.1
23. Rwanda	2014–15	1186	1.4
24. Sierra Leone	2019	2285	2.6
25. Senegal	2010–11	2771	3.2
26. Chad	2014–15	4274	4.9
27. Togo	2013–14	1977	2.3
28. Tanzania	2015–16	2944	3.4
29. Uganda	2016	4044	4.6
30. South Africa	2016	845	1.0
31. Zambia	2018	2679	3.1
32. Zimbabwe	2015	1705	1.9
All countries		87 672	100.0

### Study variables

#### Outcome variable

The outcome variables for this study were the three core indicators for infant and young child feeding practices measured as MDD, MMF and MAD. These measures were defined per the WHO's requirements^2,27^ and were categorized as ‘yes’ and ‘no’.

#### Explanatory variables

A total of 18 explanatory variables were included in the study. These variables comprised of the characteristics of the child and the mother and were grouped into individual-level, household-level, and contextual-level variables. The variables were selected based on their association with MDD, MMF, and MAD from previous literature,^9–13,20–23,28–37^ as well as their availability in the DHS dataset. For the individual-level variables, we maintained the existing coding for the sex of the child, mother's age (years), level of education, marital status, current working status and postnatal care attendance (PNC) as found in the DHS dataset. The other variables were recoded as age of child (6–8, 9–11, 12–17, 18–23 months), birth order (1, 2–4, 5+), size of the child at birth (large, average, smaller), antenatal care (ANC) attendance (none, 1–3, 4+) and place of delivery (home, health facility, other). The coding for the household-level variables included household size (small, medium, large), frequency of watching television (not at all, less than once a week, at least once a week), frequency of listening to radio (not at all, less than once a week, at least once a week), frequency of reading a newspaper/magazine (not at all, less than once a week, at least once a week), and wealth index (poorest, poorer, middle, richer, richest). The contextual-level variables consisted of place of residence (urban/rural) and geographic subregion (south, central, east, west).

### Statistical analyses

We performed both descriptive and inferential analyses using Stata version 16.0 (StataCorp, College Station, TX, USA) in this study. Descriptively, percentages with their corresponding confidence intervals (CIs) were used to present the prevalence of MDD, MMF, and MAD using forest plots. Cross-tabulation was used to determine the distribution of the outcome variables across the explanatory variables. Pearson’s χ^2^ test of independence was employed to examine the relationship between the outcome variables and the explanatory variables. All the variables that had a p-value <0.05 were regarded as statistically significant. To obtain the best combinations of variables as predictors, we employed the ‘best subset variable selection method’ to select the explanatory variables for the regression analysis. In doing this, we used the Stata command “gvselect”. A detailed description of the best selection method is provided in the literature.^38,39^ Log-likelihood, Akaike information criterion (AIC), and the Bayesian information criterion (BIC) were used to present the results of the best selection method. The combination of variables with the lowest AIC was selected for the regression analysis.

A multilevel binary logistic regression was conducted to determine the factors associated with each of the outcome variables. Five models (Models O–IV) were built to examine the predictors of the outcome variables. Model O was built to contain the outcome variable with the results indicating the variance in each of the outcome variables attributed to the clustering of the primary sample units (PSUs). Models I, II and III were built to contain the individual-level, household-level and contextual-level variables, respectively. The final model (Model IV) was fitted to include all the explanatory variables. The results of the regression analyses were presented as adjusted odds ratios (aORs) with their corresponding 95% CIs. The model fitness and comparison were checked using the AIC, with the lowest AIC showing the best-fitted model. Statistical significance was set at p<0.05. The writing of this manuscript was guided by the Strengthening Reporting of Observational Studies in Epidemiology reporting guidelines.^40^ We applied the sample weights to obtain unbiased estimates according to the DHS guidelines. Also, the Stata survey command svy was used to adjust for the complex sampling structure of the data in the χ^2^ and regression analyses.

## Results

### Prevalence of infant and young child feeding practices (MDD, MMF, and MAD) in SSA

**Figure 1.**
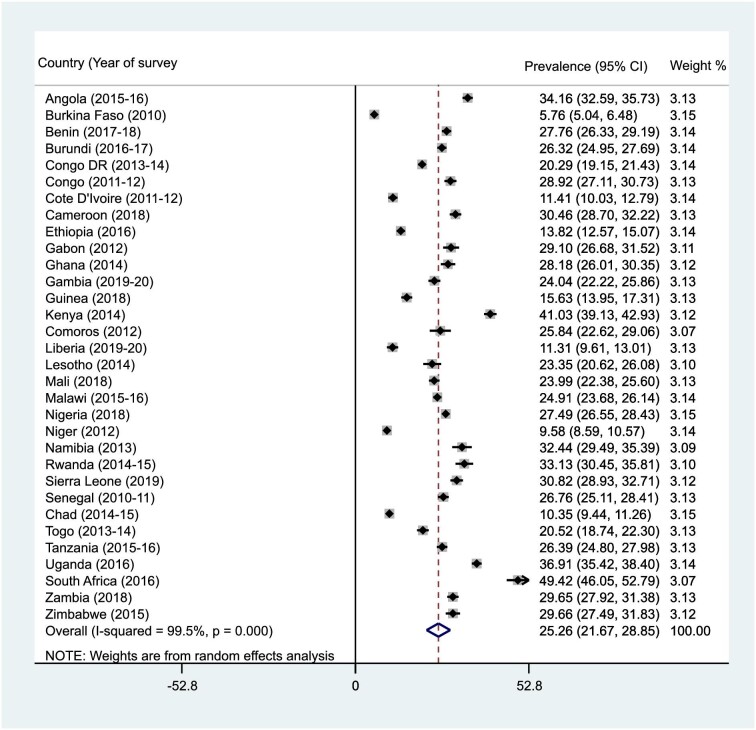
Forest plot showing the prevalence of MDD in SSA.

**Figure 2.**
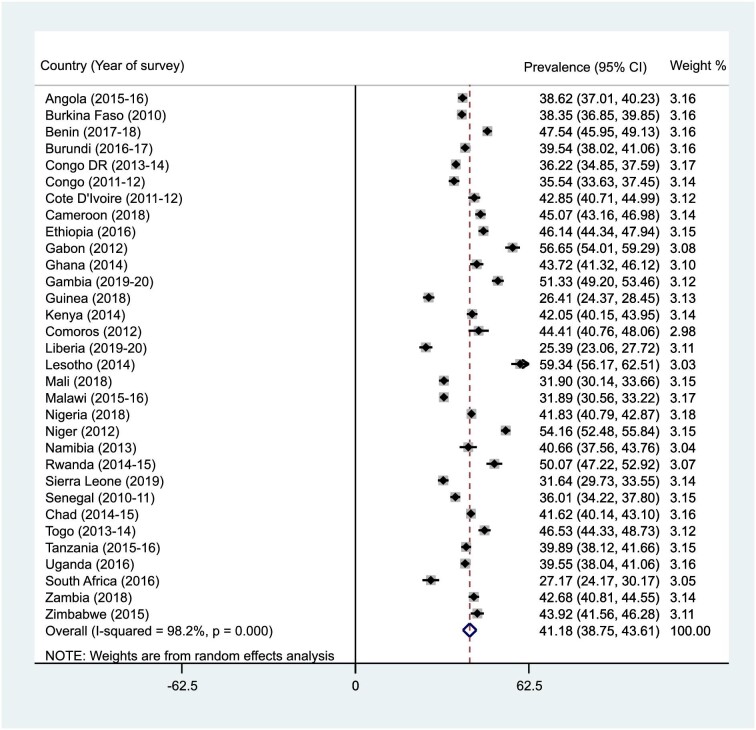
Forest plot showing the prevalence of MMF in SSA.

**Figure 3.**
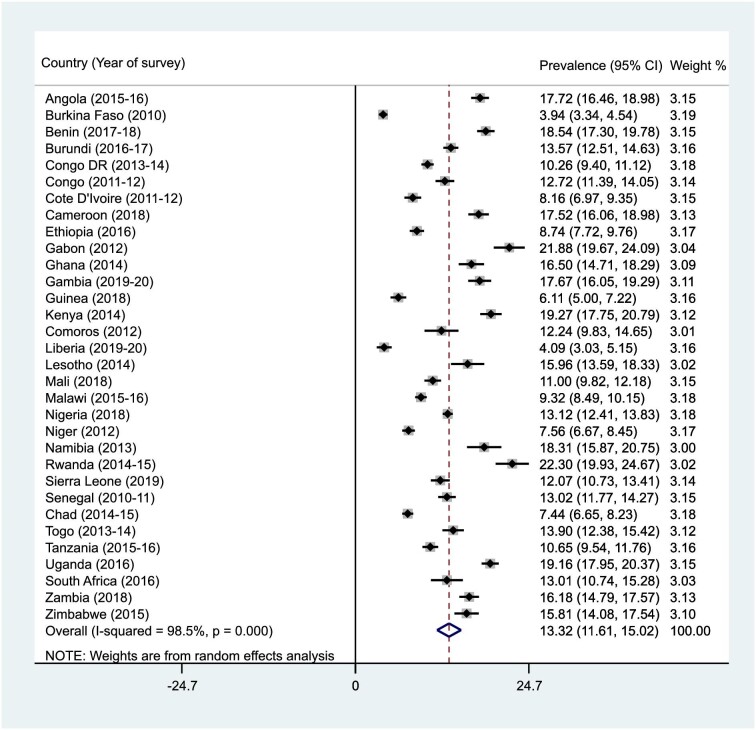
Forest plot showing the prevalence of MAD in SSA.

Figures 1–3 present the results of the prevalence of MDD, MMF, and MAD in SSA. The prevalences of MDD, MMF, and MAD in SSA were 25.3% (95% CI 21.7 to 28.9), 41.2% (95% CI 38.8 to 43.6) and 13.3% (95% CI 11.6 to 15.0), respectively. The prevalence of MDD ranged from 5.8% (95% CI 5.0 to 6.5) in Burkina Faso to 49.4% (95% CI 46.1 to 52.8) in South Africa. The study also found that while Liberia had the lowest prevalence of MMF (25.4% [95% CI 23.1 to 27.7]), Lesotho recorded the highest (59.3% [95% CI 56.2 to 62.5]). For MAD, Burkina Faso recorded the lowest prevalence (3.9% [95% CI 3.3 to 4.5]) and Rwanda had the highest (22.3% [95% CI 19.9 to 24.7]).

### Bivariate analysis of predictors of infant and young child feeding practices (MDD, MMF, and MAD) in SSA

Table 2 shows the results of the bivariate analysis of predictors of MDD, MMF, and MAD in SSA. The study found that the age of the child, birth order, size of the child at birth, maternal age, maternal education level, current working status, ANC, place of delivery, PNC, marital status, household size, frequency of watching television, frequency of listening to radio, frequency of reading a newspaper/magazine, wealth index, place of residence and geographic subregion were significantly associated with MDD and MAD. The age of the child, size of the child at birth, maternal education level, ANC, place of delivery, PNC, marital status, household size, frequency of watching television, frequency of listening to radio, frequency of reading a newspaper/magazine, wealth index, and place of residence were associated with MMF.

**Table 2.** Bivariate analysis of predictors of infant and young child feeding practices in SSA

Variables	Weighted N	Weighted %	MDD	p-Value	MMF	p-Value	MAD	p-Value
Child characteristics								
Sex of child				0.527		0.356		0.977
Male	44 296	50.5	24.5		40.6		12.8	
Female	43 376	49.5	24.3		40.9		12.8	
Age of child (months)				<0.001		<0.001		<0.001
6–8	16 253	18.5	11.1		51.0		8.5	
9–11	14 822	16.9	20.5		33.7		10.0	
12–17	31 164	35.6	27.1		39.9		13.7	
18–23	25 433	29.0	31.9		39.4		16.1	
Birth order				<0.001		0.115		<0.001
1	18 784	21.4	27.9		41.4		14.8	
2–4	42 010	47.9	25.6		40.8		13.3	
≥5	26 878	30.7	20.1		40.2		10.7	
Size of child at birth				<0.001		0.004		0.002
Large	31 034	35.4	24.5		40.9		13.0	
Average	42 405	48.4	24.9		41.2		13.1	
Smaller	14 233	16.2	22.6		39.1		11.6	
Maternal characteristics								
Mother's age (years)				<0.001		0.107		<0.001
15–19	8079	9.2	21.3		40.0		11.7	
20–24	21 866	24.9	23.5		39.9		11.9	
25–29	23 537	26.9	25.9		40.8		13.6	
30–34	17 314	19.8	25.5		41.5		13.5	
35–39	11 407	13.0	24.1		41.6		13.1	
40–44	4471	5.1	24.2		40.3		12.1	
45–49	998	1.1	19.6		42.1		10.5	
Maternal education level				<0.001		<0.001		<0.001
None	34 415	39.3	15.9		39.1		8.7	
Primary	28 165	32.1	23.7		38.9		11.8	
Secondary	22 125	25.2	34.5		44.1		18.0	
Higher	2967	3.4	54.3		52.0		31.1	
Current working status				<0.001		0.855		<0.001
No	32 670	37.3	22.0		40.8		11.6	
Yes	55 002	62.7	25.8		40.7		13.5	
ANC visits				<0.001		<0.001		<0.001
None	8814	10.1	16.3		38.0		8.7	
1–3	29 762	33.9	20.3		39.8		10.5	
≥4	49 096	56.0	28.4		41.8		15.0	
Place of delivery				<0.001		0.001		<0.001
Home	26 999	30.8	16.4		39.6		8.6	
Health facility	59 667	68.1	28.0		41.3		14.7	
Other	1006	1.1	24.6		38.0		12.2	
PNC				<0.001		<0.001		<0.001
No	51 448	58.7	22.8		38.7		11.5	
Yes	36 224	41.3	26.7		43.6		14.7	
Marital status				<0.001		<0.001		0.013
Single	6202	7.1	28.6		40.2		14.2	
Married	62 357	71.1	23.6		41.4		12.6	
Cohabiting	14 270	16.3	26.2		39.0		13.3	
Widowed	817	0.9	21.1		38.5		10.3	
Divorced	1090	1.2	21.7		38.4		11.6	
Separated	2935	3.4	26.4		38.0		13.6	
Household size				<0.001		<0.001		<0.001
Small	37 122	42.3	25.9		39.9		13.2	
Medium	38 253	43.6	23.5		41.0		12.5	
Large	12 297	14.0	22.5		42.5		12.7	
Frequency of watching television				<0.001		<0.001		<0.001
Not at all	55 299	63.1	18.5		38.5		9.4	
Less than once a week	10 399	11.9	28.0		44.0		15.4	
At least once a week	21 974	25.0	37.6		44.8		20.2	
Frequency of listening radio				<0.001		<0.001		<0.001
Not at all	39 993	45.6	19.1		38.4		9.8	
Less than once a week	17 230	19.7	24.6		42.4		13.1	
At least once a week	30 449	34.7	31.3		42.8		16.6	
Frequency of reading newspaper/magazine				<0.001		<0.001		<0.001
Not at all	74 999	85.5	21.7		39.9		11.3	
Less than once a week	779	8.3	37.2		44.5		19.5	
At least once a week	5394	6.2	45.1		47.6		24.5	
Wealth index				<0.001		<0.001		<0.001
Poorest	19 707	22.5	15.5		36.8		7.6	
Poorer	19 060	21.7	18.8		38.4		9.6	
Middle	17 827	20.3	22.6		41.1		11.8	
Richer	16 606	18.9	28.7		42.2		15.1	
Richest	14 471	16.5	41.4		47.1		22.9	
Contextual factors								
Place of residence				<0.001		<0.001		<0.001
Urban	28 367	32.4	35.3		43.2		19.0	
Rural	59 305	67.6	19.2		39.6		9.9	
Geographic subregion				<0.001		0.361		<0.001
South	2731	3.1	34.6		42.8		15.9	
Central	18 898	21.5	23.8		40.5		13.2	
East	27 490	31.4	28.5		40.4		14.1	
West	38 553	44.0	21.1		41.0		11.5	

p-values obtained from χ^2^ test.

### Predictors of infant and young child feeding practices (MDD, MMF and MAD) in SSA

Tables 3–5 show the results of the predictors of MDD, MMF, and MAD among infants and young children in SSA. The study found that those who were 18–23 months of age were more likely to have MDD (aOR 4.10 [95% CI 3.80 to 4.42]) and MAD (aOR 2.07 [95% CI 1.90 to 2.25]) but less likely to have MMF (aOR 0.61 [95% CI 0.58 to 0.64]) compared to those 6–8 months old. Again, those whose mothers had higher levels of education were more likely to have MDD (aOR 2.21 [95% CI 1.95 to 2.52]), MMF (aOR 1.37 [95% CI 1.21 to 1.54), and MAD (aOR 1.88 [95% CI 1.63 to 2.18]) compared to those who had no education. Children and infants who were delivered in a health facility were more likely to have MDD (aOR 1.16 [95% CI 1.09 to 1.24]) and MAD (aOR 1.16 [95% CI 1.07 to 1.25]) but less likely to have MMF (aOR 0.95 [95% CI 0.91 to 0.99]) compared to those who were delivered at home. Moreover, infants and young children whose mothers attended PNC were more likely to have MDD (aOR 1.06 [95% CI 1.01 to 1.11]), MMF (aOR 1.19 [95% CI 1.15 to 1.24]), and MAD (aOR 1.16 [95% CI 1.09 to 1.24]) compared to those whose mothers did not. Those whose mothers were exposed to mass media were more likely to have MDD, MMF, and MAD. Infants and young children whose mothers are of the richest wealth index were more likely to have MDD (aOR 1.67 [95% CI 1.52 to 1.84]), MMF (aOR 1.34 [95% CI 1.23 to 1.47]), and MAD (aOR 1.74 [95% CI 1.55 to 1.95]) compared to those whose mothers are of the poorest wealth index. Infants and young children whose mothers reside in rural areas were less likely to have MDD (aOR 0.74 [95% CI 0.68 to 0.79) and MAD (aOR 0.79 [95% CI 0.73 to 0.87]) compared to those who reside in urban areas.

**Table 3.** Predictors of MDD among children in SSA

		Model I,	Model II,	Model III,	Model IV,
Variable	Model O	aOR (95% CI)	aOR (95% CI)	aOR (95% CI)	aOR (95% CI)
Fixed effect results					
Age of child (months)					
6–8		1 (1.00 to 1.00)			1 (1.00 to 1.00)
9–11		2.11*** (1.94 to 2.29)			2.13*** (1.96 to 2.32)
12–17		3.21*** (2.99 to 3.44)			3.33*** (3.10 to 3.58)
18–23		3.97*** (3.69 to 4.28)			4.10*** (3.80 to 4.42)
Birth order					
1		1 (1.00 to 1.00)			1 (1.00 to 1.00)
2–4		0.90*** (0.84 to 0.95)			0.94* (0.88 to 1.00)
≥5		0.77*** (0.71 to 0.84)			0.90* (0.82 to 0.98)
Maternal education level					
None		1 (1.00 to 1.00)			1 (1.00 to 1.00)
Primary		1.50*** (1.41 to 1.59)			1.18*** (1.11 to 1.25)
Secondary		2.41*** (2.27 to 2.57)			1.54*** (1.44 to 1.66)
Higher		4.74*** (4.20 to 5.34)			2.21*** (1.95 to 2.52)
Mother's age (years)					
15–19		1 (1.00 to 1.00)			1 (1.00 to 1.00)
20–24		1.04 (0.96 to 1.13)			0.97 (0.89 to 1.05)
25–29		1.25*** (1.14 to 1.37)			1.08 (0.99 to 1.18)
30–34		1.30*** (1.18 to 1.44)			1.05 (0.95 to 1.16)
35–39		1.32*** (1.18 to 1.48)			1.05 (0.94 to 1.17)
40–44		1.48*** (1.30 to 1.68)			1.16* (1.02 to 1.32)
45–49		1.26* (1.02 to 1.55)			1.03 (0.84 to 1.27)
Current working status					
No		1 (1.00 to 1.00)			1 (1.00 to 1.00)
Yes		1.18*** (1.12 to 1.24)			1.28*** (1.22 to 1.35)
ANC visits					
None		1 (1.00 to 1.00)			1 (1.00 to 1.00)
1–3		0.90* (0.82 to 0.99)			0.84*** (0.76 to 0.92)
≥4		1.08 (0.99 to 1.18)			0.98 (0.90 to 1.07)
Place of delivery					
Home		1 (1.00 to 1.00)			1 (1.00 to 1.00)
Health facility		1.43*** (1.35 to 1.52)			1.16*** (1.09 to 1.24)
Other		1.34** (1.09 to 1.65)			1.20 (0.98 to 1.47)
PNC					
No		1 (1.00 to 1.00)			1 (1.00 to 1.00)
Yes		1.11*** (1.05 to 1.16)			1.06* (1.01 to 1.11)
Frequency of watching television					
Not at all			1 (1.00 to 1.00)		1 (1.00 to 1.00)
Less than once a week			1.31*** (1.22 to 1.41)		1.30*** (1.21 to 1.40)
At least once a week			1.56*** (1.47 to 1.66)		1.45*** (1.36 to 1.56)
Frequency of listening to radio					
Not at all			1 (1.00 to 1.00)		1 (1.00 to 1.00)
Less than once a week			1.06 (0.99 to 1.13)		1.06 (0.99 to 1.13)
At least once a week			1.26*** (1.20 to 1.34)		1.23*** (1.16 to 1.30)
Frequency of reading a newspaper/magazine					
Not at all			1 (1.00 to 1.00)		1 (1.00 to 1.00)
Less than once a week			1.49*** (1.39 to 1.59)		1.10* (1.02 to 1.19)
At least once a week			1.76*** (1.62 to 1.91)		1.24*** (1.14 to 1.36)
Wealth index					
Poorest			1 (1.00 to 1.00)		1 (1.00 to 1.00)
Poorer			1.17*** (1.10 to 1.25)		1.13*** (1.06 to 1.22)
Middle			1.36*** (1.26 to 1.46)		1.23*** (1.15 to 1.33)
Richer			1.65*** (1.53 to 1.78)		1.37*** (1.26 to 1.49)
Richest			2.35*** (2.16 to 2.56)		1.67*** (1.52 to 1.84)
Place of residence					
Urban				1 (1.00 to 1.00)	1 (1.00 to 1.00)
Rural				0.39*** (0.37 to 0.41)	0.74*** (0.68 to 0.79)
Geographic subregion					
South				1 (1.00 to 1.00)	1 (1.00 to 1.00)
Central				0.56*** (0.49 to 0.65)	0.75*** (0.65 to 0.87)
East				0.90 (0.80 to 1.02)	1.07 (0.93 to 1.22)
West				0.51*** (0.46 to 0.58)	0.67*** (0.58 to 0.77)
Random effect model					
PSU variance (95% CI)	0.17 (0.34 to 0.36)	0.12 (0.10 to 0.15)	0.12 (0.09 to 0.15)	0.14 (0.11 to 0.18)	0.12 (0.09 to 0.15)
ICC	0.05	0.04	0.03	0.04	0.03
Wald χ^2^	Reference	3327.35***	2110.03***	1259.23***	3766.06***
Model fitness					
Log-likelihood	−47 853.18	−44 214.02	−45 475.15	−46 130.23	−43 105.88
AIC	95 710.36	88 472.03	90 974.3	92 272.46	86 283.76
N	87 672	87 672	87 672	87 672	87 672
Number of clusters	1580	1580	1580	1580	1580

*p<0.05, **p<0.01, ***p<0.001.

1: reference category.

**Table 4.** Predictors of MMF among children in SSA

		Model I,	Model II,	Model III,	Model IV,
Variable	Model O	aOR (95% CI)	aOR (95% CI)	aOR (95% CI)	aOR (95% CI)
Fixed effect results					
Age of child (months)					
6–8		1 (1.00 to 1.00)			1 (1.00 to 1.00)
9–11		0.48*** (0.45 to 0.51)			0.47*** (0.45 to 0.51)
12–17		0.63*** (0.60 to 0.66)			0.63*** (0.60 to 0.66)
18–23		0.61*** (0.58 to 0.64)			0.61*** (0.58 to 0.64)
Birth order					
1		1 (1.00 to 1.00)			1 (1.00 to 1.00)
2–4		0.96 (0.91 to 1.02)			0.97 (0.92 to 1.03)
≥5		0.94 (0.88 to 1.02)			0.94 (0.88 to 1.02)
Size of child at birth					
Large		1 (1.00 to 1.00)			1 (1.00 to 1.00)
Average		1.02 (0.98 to 1.06)			1.02 (0.98 to 1.06)
Smaller		0.95* (0.90 to 1.00)			0.95 (0.90 to 1.00)
Maternal education level					
None		1 (1.00 to 1.00)			1 (1.00 to 1.00)
Primary		1.03 (0.98 to 1.08)			1.00 (0.95 to 1.05)
Secondary		1.27*** (1.20 to 1.34)			1.14*** (1.08 to 1.21)
Higher		1.67*** (1.49 to 1.86)			1.37*** (1.21 to 1.54)
Mother's age (years)					
15–19		1 (1.00 to 1.00)			1 (1.00 to 1.00)
20–24		1.01 (0.94 to 1.08)			1.00 (0.93 to 1.07)
25–29		1.04 (0.97 to 1.13)			1.01 (0.94 to 1.10)
30–34		1.08 (0.99 to 1.18)			1.03 (0.95 to 1.12)
35–39		1.12* (1.02 to 1.23)			1.06 (0.97 to 1.17)
40–44		1.08 (0.97 to 1.21)			1.03 (0.92 to 1.15)
45–49		1.21* (1.02 to 1.45)			1.17 (0.98 to 1.39)
Marital status					
Single		1 (1.00 to 1.00)			1 (1.00 to 1.00)
Married		1.15*** (1.07 to 1.24)			1.17*** (1.08 to 1.26)
Cohabiting		1.02 (0.94 to 1.10)			1.04 (0.96 to 1.13)
Widowed		1.03 (0.85 to 1.26)			1.09 (0.90 to 1.32)
Divorced		1.04 (0.88 to 1.23)			1.07 (0.90 to 1.27)
Separated		0.98 (0.88 to 1.10)			1.01 (0.90 to 1.12)
Place of delivery					
Home		1 (1.00 to 1.00)			1 (1.00 to 1.00)
Health facility		1.0 (0.95 to 1.04)			0.95* (0.91 to 0.99)
Other		0.91 (0.76 to 1.08)			0.89 (0.74 to 1.07)
PNC					
No		1 (1.00 to 1.00)			1 (1.00 to 1.00)
Yes		1.21*** (1.16 to 1.26)			1.19*** (1.15 to 1.24)
Frequency of watching television					
Not at all			1 (1.00 to 1.00)		1 (1.00 to 1.00)
Less than once a week			1.13*** (1.07 to 1.20)		1.14*** (1.08 to 1.21)
At least once a week			1.09** (1.04 to 1.16)		1.09** (1.03 to 1.15)
Frequency of listening to radio					
Not at all			1 (1.00 to 1.00)		1 (1.00 to 1.00)
Less than once a week			1.08** (1.03 to 1.14)		1.07** (1.02 to 1.13)
At least once a week			1.07** (1.02 to 1.12)		1.06* (1.01 to 1.11)
Frequency of reading a newspaper/magazine					
Not at all			1 (1.00 to 1.00)		1 (1.00 to 1.00)
Less than once a week			1.07* (1.00 to 1.15)		1.01 (0.94 to 1.09)
At least once a week			1.17*** (1.08 to 1.28)		1.09 (1.00 to 1.20)
Household size					
Small			1 (1.00 to 1.00)		1 (1.00 to 1.00)
Medium			1.07*** (1.03 to 1.11)		1.07*** (1.03 to 1.12)
Large			1.12*** (1.05 to 1.19)		1.13*** (1.06 to 1.20)
Wealth index					
Poorest			1 (1.00 to 1.00)		1 (1.00 to 1.00)
Poorer			1.05* (1.00 to 1.11)		1.05 (1.00 to 1.11)
Middle			1.15*** (1.09 to 1.22)		1.15*** (1.08 to 1.21)
Richer			1.18*** (1.11 to 1.25)		1.17*** (1.10 to 1.25)
Richest			1.38*** (1.28 to 1.49)		1.34*** (1.23 to 1.47)
Place of residence					
Urban				1 (1.00 to 1.00)	1 (1.00 to 1.00)
Rural				0.84*** (0.80 to 0.89)	1.06 (1.00 to 1.13)
Geographic subregion					
South				1 (1.00 to 1.00)	1 (1.00 to 1.00)
Central				0.91 (0.81 to 1.02)	1.05 (0.93 to 1.19)
East				0.95 (0.85 to 1.05)	1.03 (0.92 to 1.16)
West				0.94 (0.85 to 1.05)	0.98 (0.88 to 1.10)
Random effects model					
PSU variance (95% CI)	0.07 (0.06 to 0.09)	0.07 (0.06 to 0.09)	0.07 (0.06 to 0.09)	0.07 (0.06 to 0.09)	0.07 (0.06 to 0.09)
ICC	0.02	0.02	0.02	0.02	0.02
Wald χ2	Reference	893.35***	288.98***	45.49***	1069.89***
Model fitness					
Log-likelihood	−58 284.58	−57 555.70	−58 085.86	−58 322.89	−57 401.81
AIC	116 773.2	115 163.4	116 199.7	116 657.8	114 887.6
N	87 672	87 672	87 672	87 672	87 672
Number of clusters	1580	1580	1580	1580	1580

aOR = adjusted odds ratios; CI = Confidence Interval; * *p* < 0.05, ** *p* < 0.01, *** *p* < 0.001; 1 = Reference category; PSU = Primary Sampling Unit; ICC = Intra-Class Correlation; AIC = Akaike's Information Criterion

**Table 5.** Predictors of an MAD among children in SSA

		Model I,	Model II,	Model III,	Model IV,
Variable	Model O	aOR (95% CI)	aOR (95% CI)	aOR (95% CI)	aOR (95% CI)
Fixed effect results					
Age of child (months)					
6–8		1(1.00 to 1.00)			1 (1.00 to 1.00)
9–11		1.17** (1.06 to 1.29)			1.17** (1.06 to 1.29)
12–17		1.73*** (1.59 to 1.87)			1.75*** (1.62 to 1.90)
18–23		2.05*** (1.88 to 2.23)			2.07*** (1.90 to 2.25)
Birth order					
1		1 (1.00 to 1.00)			1 (1.00 to 1.00)
2–4		0.90* (0.83 to 0.98)			0.93 (0.86 to 1.01)
≥5		0.81*** (0.73 to 0.91)			0.88* (0.79 to 0.98)
Maternal education level					
None		1 (1.00 to 1.00)			1 (1.00 to 1.00)
Primary		1.30*** (1.20 to 1.40)			1.10* (1.01 to 1.19)
Secondary		1.98*** (1.84 to 2.14)			1.37*** (1.26 to 1.49)
Higher		3.55*** (3.11 to 4.05)			1.88*** (1.63 to 2.18)
Mother's age (years)					
15–19		1 (1.00 to 1.00)			1 (1.00 to 1.00)
20–24		0.96 (0.87 to 1.07)			0.92 (0.83 to 1.03)
25–29		1.17* (1.04 to 1.31)			1.04 (0.92 to 1.17)
30–34		1.20** (1.06 to 1.36)			1.01 (0.89 to 1.15)
35–39		1.25** (1.09 to 1.44)			1.04 (0.91 to 1.19)
40–44		1.24** (1.05 to 1.47)			1.02 (0.86 to 1.21)
45–49		1.18 (0.90 to 1.55)			1.02 (0.78 to 1.33)
ANC visits					
None		1 (1.00 to 1.00)			1 (1.00 to 1.00)
1–3		0.87* (0.77 to 0.99)			0.82** (0.72 to 0.93)
≥4		1.02 (0.91 to 1.15)			0.93 (0.83 to 1.05)
Place of delivery					
Home		1 (1.00 to 1.00)			1 (1.00 to 1.00)
Health facility		1.38*** (1.28 to 1.49)			1.16*** (1.07 to 1.25)
Other		1.26 (0.91 to 1.73)			1.17 (0.85 to 1.60)
PNC					
No		1 (1.00 to 1.00)			1 (1.00 to 1.00)
Yes		1.20*** (1.13 to 1.28)			1.16*** (1.09 to 1.24)
Current working status					
No		1 (1.00 to 1.00)			1 (1.00 to 1.00)
Yes		1.14*** (1.08 to 1.21)			1.21*** (1.14 to 1.28)
Frequency of watching television					
Not at all			1 (1.00 to 1.00)		1 (1.00 to 1.00)
Less than once a week			1.35*** (1.24 to 1.48)		1.31*** (1.20 to 1.44)
At least once a week			1.48*** (1.37 to 1.60)		1.33***(1.22 to 1.45)
Frequency of listening to radio					
Not at all			1 (1.00 to 1.00)		1 (1.00 to 1.00)
Less than once a week			1.07 (0.99 to 1.16)		1.07 (0.99 to 1.16)
At least once a week			1.23*** (1.15 to 1.32)		1.21*** (1.14 to 1.30)
Frequency of reading a newspaper/magazine					
Not at all			1 (1.00 to 1.00)		1 (1.00 to 1.00)
Less than once a week			1.34*** (1.23 to 1.46)		1.08 (0.98 to 1.18)
At least once a week			1.55*** (1.40 to 1.71)		1.18** (1.05 to 1.31)
Household size					
Small			1 (1.00 to 1.00)		1 (1.00 to 1.00)
Medium			1.01 (0.95 to 1.07)		1.09** (1.03 to 1.16)
Large			0.98 (0.90 to 1.08)		1.19*** (1.09 to 1.31)
Wealth index					
Poorest			1 (1.00 to 1.00)		1 (1.00 to 1.00)
Poorer			1.20*** (1.10 to 1.31)		1.16** (1.06 to 1.27)
Middle			1.39*** (1.28 to 1.52)		1.28*** (1.17 to 1.41)
Richer			1.67*** (1.52 to 1.83)		1.42*** (1.29 to 1.57)
Richest			2.33*** (2.10 to 2.58)		1.74*** (1.55 to 1.95)
Place of residence					
Urban				1 (1.00 to 1.00)	1(1.00 to 1.00)
Rural				0.44*** (0.41 to 0.46)	0.79*** (0.73 to 0.87)
Geographic subregion					
South				1 (1.00 to 1.00)	1(1.00 to 1.00)
Central				0.78** (0.66 to 0.93)	1.05 (0.88 to 1.26)
East				1.04 (0.89 to 1.22)	1.24* (1.04 to 1.47)
West				0.72*** (0.62 to 0.85)	0.91 (0.76 to 1.08)
Random effects model					
PSU variance (95% CI)	0.16 (0.13 to0.21)	0.13 (0.11 to 0.17)	0.14 (0.11 to 0.17)	0.15 (0.12 to 0.19)	0.14 (0.11 to 0.17)
ICC	0.05	0.04	0.04	0.04	0.04
Wald χ^2^	Reference	1304.69***	1260.75***	687.02***	1828.01***
Model fitness					
Log-likelihood	−32 977.28	−31 596.31	−31 712.36	−32 205.64	−31 042.86
AIC	65 958.56	63 236.62	63 452.72	64 423.28	62 161.72
N	87 672	87 672	87 672	87 672	87 672
Number of clusters	1580	1580	1580	1580	1580

*p<0.05, **p<0.01, ***p<0.001.

1: reference category.

## Discussion

This study examined the prevalence and predictors of MDD, MMF, and MAD in 32 countries in SSA. The prevalence of MDD, MMF, and MAD in SSA were 25.3%, 41.2%, and 13.3%, respectively. The low prevalence of MDD, MMF and MAD recorded in this study are similar to what was recorded in 80 LMICs,^41^ 49 LMICs^42^ and 48 LMICs.^43^ A possible reason for the similarities in the findings could be attributed to the similarities in the socio-economic status of the LMICs and the employment of a large sample size.^42,43^ Variations were also detected for the prevalence of infant and child feeding practices among the studied countries. The prevalence of MDD ranged from 5.8% in Burkina Faso to 49.4% in South Africa. This could be attributed to the lower proportion of women reaching the MDD in Burkina Faso.^31^ The study also found that while Liberia had the lowest (25.4%) prevalence of MMF, Lesotho recorded the highest (59.3%). The level of poverty in Liberia could also account for this finding.^28^ For MAD, Burkina Faso recorded the lowest prevalence (3.9%) while Rwanda had the highest (22.3%), probably because of the mothers’ low level of perceived self-efficacy to provide the daily required food groups for their children.^44^

The prevalence of MDD, MMF, and MAD vary across different countries in SSA. For example, a study conducted in Ghana found the prevalence of MDD to be 35.6%,^45^ whereas a survey in Ethiopia found a lower prevalence of MDD, which was 10.8%.^22^ In Malawi, a 27.7% prevalence of MDD was found. For MMF, an Ethiopian survey found the prevalence to be 44.7%,^22^ whereas a study in Nigeria reported a prevalence of 56%.^46^ The prevalence of MAD was found to be as low as 7.0% in a study conducted in Ethiopia,^47^ while in Nigeria, a study reported the prevalence of MAD to be 8.0%.^46^

Studies conducted in different sub-Saharan African countries have reported the factors associated with MDD to include the mother's education, wealth quintile, urban residence, home gardening, media exposure, mother's knowledge of dietary diversity, employment status, household assets, and optimal household water access.^11,22,23,48,49^ On the other hand, factors such as child age, parity of the mother, wealth quintile, mother's education level, maternal age, mother watching television, size of the baby, mode of delivery, and health service contact have been reported to be associated with MMF in different countries in SSA.^12,28,41,46^ The factors that are associated with MAD across different parts of SSA include ANC visits, mother's education level, household wealth quintile, age of the child, sex of the child, mother's media usage, mother's working status, and birth interval.^28,46^

The study found that infants and young children who were 18–23 months of age were more likely to have MDD and MAD. This finding is similar to the findings of previous studies.^22,44,45^ An explanation for these findings could be a result of an increase in the consumption of other food groups as children age.^22,44^ However, those who were 18–23 months were less likely to have MMF. It is possible the reduction in exclusive breastfeeding practices as children grow could have influenced their likelihood of receiving the recommended MMF compared to their younger counterparts.^50^

Similar to the findings of previous studies,^23,51–53^ this study found that infants and young children whose mothers had higher education levels were more likely to have MDD, MMF, and MAD. This finding could be that women who are more educated are better equipped with knowledge about providing their children with the appropriate complementary foods they require, increasing their likelihood of adequately feeding their children.^23,51,53^ This finding could be because women who are more educated are mostly employed, which suggests that they may have a greater ability to afford the variety of required food groups their children need for optimum growth and development.^23,51,53^

Corroborating the findings of previous studies,^53–55^ the study found that infants and young children who were delivered in a health facility were more likely to have MDD and MAD. A potential explanation for this finding could be that women who deliver in a health facility receive some education about complementary feeding practices, increasing their likelihood of adequately feeding their children.^53,54^ However, those who were delivered in a health facility were less likely to have MMF. This finding was also confirmed by a previous study in Tanzania.^53^ A possible reason for this could be that mothers continuously practice exclusive breastfeeding as children grow instead of introducing complementary foods.^53^

Moreover, infants and young children whose mothers attended PNC were more likely to have MDD, MMF and MAD. The finding of this study is similar to the findings of previous studies.^23,53,55^ Women who frequently visited a health facility could have benefited from the education given to mothers regarding choosing and providing complementary feeding for their children to aid their development while reducing the occurrence of preventable diseases.^53,55^

Similar to the findings of other studies,^22,51,56^ our study found that infants and young children whose mothers were exposed to mass media were more likely to have MDD, MMF and MAD. Our finding could imply that women who are exposed to mass media are educated through this means regarding the appropriate feeding practices available to them, increasing their likelihood of adequately feeding their children.^22,51^

Corroborating the findings of previous studies,^21–23,51–53^ this study found that infants and young children whose mothers are of the richest wealth index were more likely to have MDD, MMF and MAD. Our finding could be the fact that wealthy households are able to afford and provide a variety of the required food groups to facilitate proper growth and development of young children, increasing their likelihood of being adequately fed.^21,22,52^

Similar to the findings of previous studies,^51,56^ this study found that infants and young children whose mothers reside in rural areas were less likely to have MDD and MAD. Our finding may be due to economic reasons, given that rural areas may be less developed and individuals living there may not be as educated and financially able, which may reduce their likelihood of adequately feeding their children.^51,56^

## Strengths and limitations

The use of a relatively large sample size of nationally representative samples from several countries makes the findings of the study more generalizable to the study populations used in this study. However, the study has some limitations. First, the study was cross sectional, thus causal interpretations of the findings are limited. Again, the variables used were self-reported, thus respondents might have under- or overreported the feeding practices of their children, which could influence the findings.

### Conclusions

The study found that the prevalence of MDD, MMF, and MAD among infants and young children in 32 countries in SSA remains low. Variations in the prevalence of feeding practices among infants and young children in the studied countries were also identified. The study also identified the factors that are associated with MDD, MMF, and MAD. Public health interventions aimed at improving complementary feeding practices among infants and young children in SSA should focus on the factors identified in this study. Regarding the poor state of complementary feeding practices for infants and young children, the study recommends that regional and national policies on food and nutrition security and maternal and child nutrition and health follow the internationally recommended guidelines in promoting, protecting and supporting age-appropriate complementary foods and feeding practices for infants and young children.

## Data Availability

The dataset for the 32 countries used is freely available from https://dhsprogram.com/data/available-datasets.cfm.
